# The native human glomerulus features a slit diaphragm resembling a densely interwoven fishnet

**DOI:** 10.1172/jci.insight.200658

**Published:** 2026-01-09

**Authors:** Deborah Moser, Alexandra N. Birtasu, Lilli Skaer, Pauline Roth, Lisa Rehm, Mike Wenzel, Julia Bein, Jens Köllermann, Mbuso S. Mantanya, Felix K.H. Chun, Margot P. Scheffer, Achilleas S. Frangakis

**Affiliations:** 1 Buchmann Institute for Molecular Life Sciences, Goethe University Frankfurt, Frankfurt, Germany.; 2 Department of Urology and; 3 Department of Pathology, Goethe University Hospital Frankfurt, Frankfurt, Germany.

**Keywords:** Cell biology, Nephrology, Structural biology

## Abstract

The native human glomerulus features a slit diaphragm resembling a densely interwoven fishnet

**To the Editor:** A comprehensive understanding of the molecular architecture of the slit diaphragm (SD), a critical component of the glomerular filtration barrier, is essential in elucidating renal filtration physiology and the mechanisms underlying kidney disease (1, 2). Disruption of the SD is a hallmark of all forms of glomerulopathy. Our recent work, based on our ability to analyze the native unstained specimen (see Birtasu et al. regarding other preparations and imaging modalities) demonstrated that the SD architecture in mice and Drosophila resembles a fishnet, with species-specific structural adaptations (3, 4). Here, we visualize the native in situ architecture of the human SD at an unprecedented resolution through cryo-electron tomography (cryo-ET) of human glomeruli.

Kidney tissue was obtained from an adult patient undergoing a right nephrectomy for renal cell carcinoma (Figure 1A). For cryo-ET, nontumorous renal cortex was sampled from a region distal to the tumor margin, representative of healthy kidney tissue, from which we extracted 18 glomeruli (Figure 1B). For processing tissue of this size for cryo-ET, particularly native human tissue, we implemented a dedicated preparation pipeline combining high-pressure freezing (without the use of any chemicals or heavy metal staining), focused-ion beam milling, and cryo-ET (Supplemental Figure 1 and Supplemental Methods; supplemental material available online with this article; https://doi.org/10.1172/jci.insight.200658DS1). In the micrographs equidistant strands (~9 nm apart) spanning the foot processes (fp) can be discerned (Supplemental Figure 2). In the tomograms the SD spans adjacent fp, forming a fine, web-like mesh of crisscrossing strands across the extracellular space, resembling a fishnet (Figure 1C and Supplemental Figure 3). The underlying actin cytoskeleton appears bundled along the axis of the fp and increasingly branched and loosely organized near the membranes (Figure 1D). A total of 62 SD segments were averaged, yielding a map of the human SD at a resolution of ~4.5 nm (Figure 1E), revealing crisscrossing strands intersecting at ~90°, forming a fishnet pattern spanning the ~44 nm extracellular space between fp. Using crystallographic structures (5) and AlphaFold predictions (IDs: AF-Q9QZS7-F1, AF-Q80W68-F1), we built an atomic model of the Nephrin-Neph1 heterodimer, the main constituents of the SD that belong to the immunoglobulin (Ig) superfamily and consist of 10 and 5 Ig domains, respectively (6). The heterodimers were then assembled into a model of the human SD that reflected the densities of the cryo-ET map (Figure 1F and Supplemental Figure 4A).

The SD has overall the same fishnet architecture in humans as in mice (3) and Drosophila (4), while it is more densely woven in humans with individual strands crisscrossing to form a tight mesh (Figure 1F). In both humans and mice, the same number of Ig domains exist, though there is a genetic variation between the Ig domains allowing for different interaction sites, while retaining the overall architecture (3, 4). The heterodimers in humans are spaced ~9 nm apart, which is closer than in mice (12.3 nm) or Drosophila (15 nm), resulting in 7 crossing points per heterodimer compared with 4 crossing points in mice and 3 in Drosophila (Supplemental Figure 4C). This tighter organization in the human SD produces smaller structural holes of ~5 × 5 nm versus ~7 × 7 nm in mice and ~12 × 12 nm in Drosophila (Figure 1F and Supplemental Figure 4B). The additional interaction points in the human SD suggest greater heterodimer stability, reflecting a functional specialization and adaptation of the human glomerular filtration barrier.

Together, we show that the human SD resembles a finely webbed fishnet, which is evolutionarily conserved in Drosophila and mouse. The densely interwoven human SD likely has immediate consequences for the mechanical stability and the turnover of individual Nephrin/Neph1 molecules. While the active filtration role of the SD is not ultimately clarified, we hypothesize that the permselectivity in humans, in particular at homeostatic imbalance, will be higher than in mice or Drosophila, as the passage of filtrates is more restricted (Supplemental Figure 4B) (7). Importantly, our ability to analyze native human tissue at a nanometer resolution enables opportunities for the in situ investigation of disease mechanisms and therapeutic development.

Caveats of our study include restriction to a single patient. While variations between individuals are likely due to genetic variations, the SD fishnet architecture will be similar in other individuals. The fishnet architecture of the SD is unambiguous. However, model generation of the human SD relies on better resolved studies. Thus, while all future models of the human SD will look like fishnets, insights into the exact molecular arrangement will improve with more data.

## Funding support

- DFG for Research Training Group iMOL (GRK 2566/1) for DM, ANB, LS, and MSM.
- DFG for MPS (FR 1653/14-1).

## Supplementary Material

Supplemental data

## Figures and Tables

**Figure 1:**
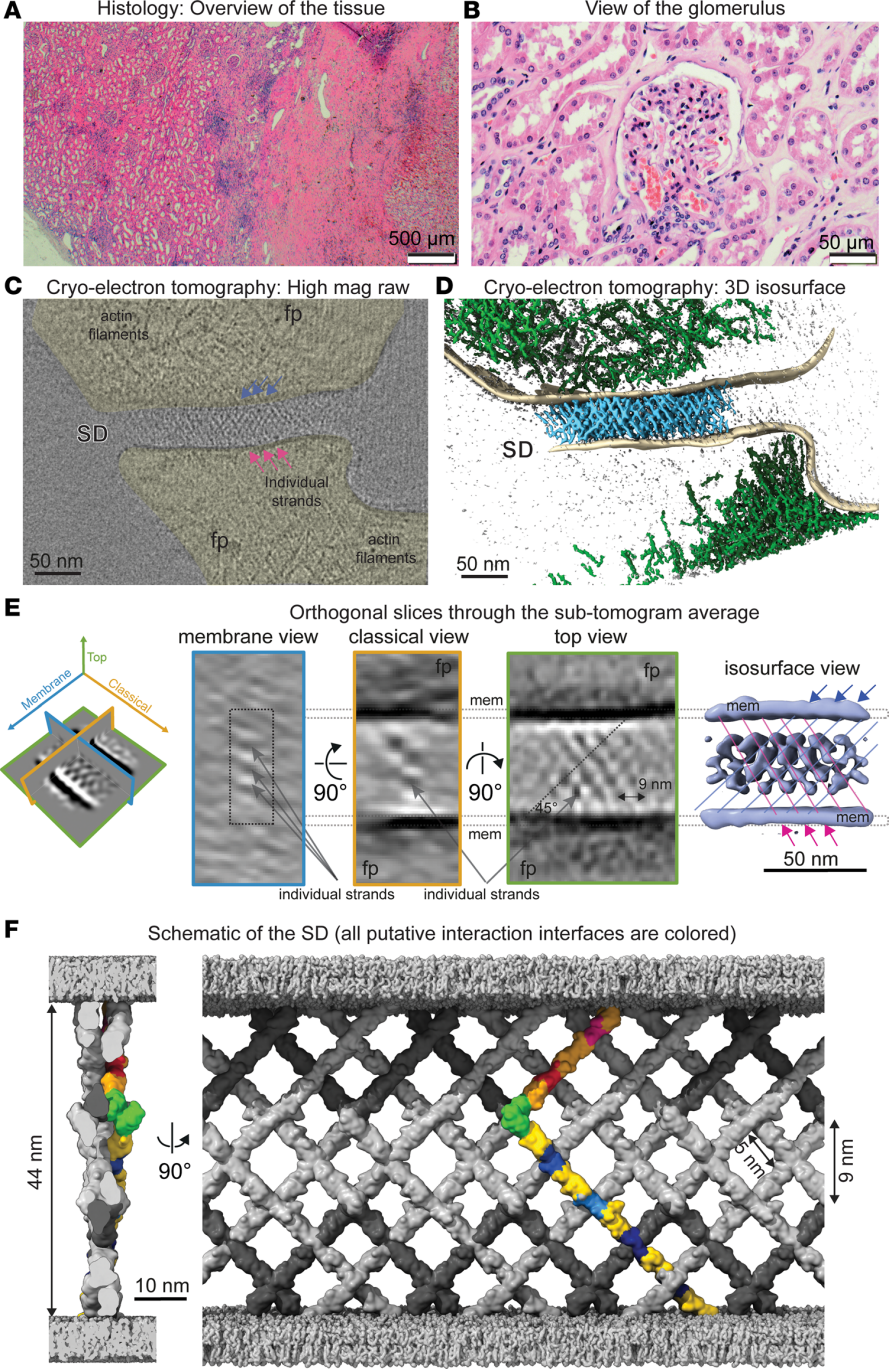
Architecture of the human glomerular filtration system. (**A**) Histology image of a human nephrectomized kidney biopsy. (**B**) Glomerulus image showing normal pathology. (**C**) Slice through a cryo-electron tomogram of the filtration system. Foot processes (fp; yellow) contain actin filaments. The top view (en face) of the SD shows crisscrossing strands forming a fishnet; strands spanning the membrane indicated by blue and pink arrows. (**D**) Isosurface representation of the cryo-electron tomogram (in **C**) displaying the SD (in blue) from the top view spanning the 2 plasma membranes (in beige) of the fp. Large areas of the fp are filled with branched actin (in green). (**E**) Three orthogonal views — membrane view (blue frame), classical view (orange frame), and top view (green frame) — of computational sections through the sub-tomogram average. Crisscrossing densities span the plasma membranes (“mem”) of fp. Membrane and classical views show individual strand sections (dots), and top and isosurface views reveal a fishnet pattern with the strands crossing at ~90°. The isosurface view of the sub-tomogram average shows the fishnet pattern with internal structural holes. Diagonal light blue and pink lines indicate crossing strands, highlighting the fishnet pattern. Lines are spaced 9 nm apart. (**F**) Idealized molecular model of the human SD shown in 2 orthogonal views. The Nephrin-Neph1 heterodimer was used as a unit cell and placed in 1-dimensional crystal packing. One heterodimer is highlighted in gold with the putative interfaces shown in various colors. All other heterodimers are shown in gray (Nephrin in light gray and Neph1 in dark gray). Each heterodimer is predicted to interact with 6 other heterodimers.

## Version 1 01/09/2026

Electronic publication
