# Supplementary material for: The native human glomerulus features a slit diaphragm resembling a densely interwoven fishnet
Source: JCI Insight. 2026 Jan 9;11(1):e200658. doi: 10.1172/jci.insight.200658 (PMC12890517; doi:10.1172/jci.insight.200658)
Supplement: Supplemental data [file jciinsight-11-200658-s006.pdf]

Supplemental Data

Materials & Methods

Isolation of the human glomeruli

Isolation of glomeruli from nephrectomized human kidney tissue was performed according to an adapted isolation protocol (6). All steps were performed at 4°C or on ice. In brief, a small tissue piece was harvested from nephrectomized human kidney tissue and minced into small pieces (~1 mm^3) in Hanks' Balanced Salt Solution (HBSS, #2323615, Gibco, Thermo Fisher Scientific Inc., Waltham, MA, USA). Minced tissue pieces were gently pressed with a wooden tongue depressor (Glaswarenfabrik Karl Hecht, Sondheim vor der Rhön, Germany), first through an HBSS pre-wetted 150 µm cell strainer and then a 100 µm cell strainer (PluriStrainer, PluriSelect Life Sciences, Leipzig, Germany). The retained sample was rinsed into a centrifuge tube and centrifuged at 115 *x g* for 5 min at 4°C. The pellet was resuspended in 100 µl CellBrite Steady Membrane Stain 550 (#30107, Biotium, Fremont, CA, USA) in HBSS (1:1000) and incubated for 25 min, followed by vitrification by high-pressure freezing.

Sample vitrification by high-pressure freezing

Vitrification of the sample was based on a modified preparation protocol (7). Planchets (type B, Wohlwend, Sennwald, Switzerland) were coated with cera alba (1% w/v in diethyl ether) prior to freezing. Formvar-coated grids (copper/palladium, 75-mesh parallel single bar; G2018D, Plano, Wetzlar, Germany) were placed on a planchet, and 3 µl of isolated glomeruli and tubular fragments mixed with Ficoll PM 400 (20% v/v in HBSS) (Sigma-Aldrich, St. Louis, MO, USA) were applied before the sandwich was completed by adding a second planchet. The sample was then high-pressure frozen using an HPM-010 (Abra Fluid, Widnau, Switzerland). Samples were kept at a temperature low enough to prevent ice crystallization and to guarantee that both the sample and the ice remained in a vitreous state.

Cryogenic confocal laser scanning microscopy

High-pressure frozen grids were imaged at -196°C by confocal laser scanning microscopy (cryo-CLSM) (CMS196 cryo-stage, Linkam, Salfords, United Kingdom; LSM700, Carl Zeiss, Jena, Germany). Optical configurations were adjusted to capture the autofluorescence of the glomeruli in the green channel (excitation wavelength of 488 nm), the membrane stain in the red channel (excitation wavelength of 555 nm), and the reflection from the grids in the far-red channel (excitation wavelength of 639 nm). Images were acquired with a 5x/ NA 0.16 objective. Data acquisition was performed with Zeiss ZEN 2009 (blue) v2.1.

Cryo-focused ion beam milling

The high-pressure-frozen electron microscopy (EM) grids were clipped into cryo-focused ion beam (FIB) autogrids (#1205101, Thermo Fisher Scientific Inc.) and loaded into an EM grid holder under liquid nitrogen using an EM vacuum cryo-transfer system

VCT500 loading station (Leica Microsystems, Wetzlar, Germany). The sample was transferred to an ACE600 high-vacuum sputter coater (Leica Microsystems) for platinum coating (8 nm) using an EM VCT500 transfer shuttle via a VCT dock (Leica Microsystems). The sample holder was subsequently transferred into a FIB scanning electron microscope (SEM), i.e. a dual-beam instrument (Helios 600i Nanolab, Thermo Fisher Scientific Inc.) that is equipped with a band-cooled cryo-stage equilibrated at -163°C (Leica Microsystems). The EM grids were imaged using the SEM (3 kV, 0.21 pA) and the FIB source (gallium ion source, 30 kV, 18 pA). An overview SEM image was acquired at 90° incident angle. An organometallic platinum layer of a few microns thickness was deposited with the gas injection system. Regions of interest were identified by correlating the cryo-CLSM images with the SEM images using Bigwarp. Localized positions were milled by applying a specific stress-relief gap for waffled grids. The net incident angle varied between 23° and 28°. The following FIB currents were used: 9.4 – 45 nA (trenching), 2.5 nA stepwise down to 83 pA (thinning), 240 pA (notch milling), and 33 pA (polishing). During thinning steps, a second platinum sputtering was done (8 nm), followed by deposition of an organometallic platinum layer that was a few microns thick.

Cryogenic transmission electron microscopy imaging and cryo-electron tomography

Cryo-FIB milled lamellae (2 glomeruli in total) were imaged using a Titan Krios cryogenic transmission electron microscope (cryo-TEM; Thermo Fisher Scientific Inc.) operating at 300 kV in nanoprobe EFTEM mode, equipped with an X-FEG field emission-gun, a GIF Quantum post-column energy filter operating in zero-loss mode and a K3 direct electron detector (Gatan Inc., Pleasanton, CA, USA). Low-magnification images (×11500) of the lamellae were acquired at an angle between 23° and 28° to match the lamella pre-tilt angle induced by FIB milling (calibrated pixel size 1.642 nm/pix, –50 µm defocus).

Tilt series were recorded at a nominal magnification of ×33,000 (calibrated pixel size 1.34 Å/pix) in super-resolution and dose fractionation mode. The cumulative total dose per tomogram was between 130 e− Å-2, and 150 e− Å-2, and the tilt series covered an angular range from −66° to +66° in reference to the lamella pre-tilt, with an angular increment of 2° to 3° and the nominal defocus set to –5 µm. The complete preparation and recording pipeline are presented in Supplementary figure S1.

Image processing and Artia-Wrapper sub-tomogram averaging

The movie stacks were aligned to compensate for beam-induced movement using the MotionCor2 wrapper within RELION-5 (8), and the contrast transfer function subsequently estimated using CTFFIND4 v4.1.1426. The tilt series were then aligned using IMOD v4.11.24 patch tracking (9). The tomographic reconstructions were performed by various algorithms such as simultaneous algebraic reconstruction technique for high-contrast and weighted back-projection for sub-tomogram averaging (10).

Tomogram segmentation was performed using Dragonfly (v. 2024.1 for Windows), and the smoothness of the segmentations was improved using mean curvature motion (11) (https://github.com/FrangakisLab/mcm-cryoet). MATLAB scripts were used to measure the distance between two adjacent podocyte membranes and the periodicity of the densities forming the SD by cross-correlation (MATLAB 2023a, 2023 and MATLAB 2024a, 2024; The MathWorks, Natick, MA, USA, https://github.com/FrangakisLab/membraneDistanceMeasurement).

In the tomographic reconstructions, oblique planes were placed on the SD based on orientation of the glomerular basement membrane, and points on individual strands were selected on the plane using ArtiaX (12) implemented in UCSF ChimeraX (13). A total of 62 sub-tomograms (box size 128 pix, pixel size 5.36 Å) were extracted. All sub-tomograms showed a fishnet pattern but with a strong missing wedge, leading to a very strong signal anisotropy. Consequently, sub-tomogram averaging was used more to confirm the similarity of the data to the murine and nephrocyte models than for structural purposes.

Slit diaphragm modelling of the Nephrin-Neph1 heterodimers

The molecular model of Nephrin-Neph1 heterodimers was generated from predictions available from the AlphaFold Protein Structure Database (14, 15) (IDs: AF-Q9QZS7-F1, AF-Q80W68-F1) with the help of Coot, since no full-length structure is available to date. The unstructured regions at the N- and C-termini with low predicted confidence were removed (Nephrin: amino acids 1–29 and 1038–1241; Neph1: 1–19 and 490–757). The angle between Nephrin and Neph1 was estimated from the cryo-ET map at 90°. Nephrin and Neph1 were then assembled into a heterodimer based on the crystal structure of the SYG-2-SYG-1 heterodimer (ID: 4OFY) (5). The heterodimers were then placed according to the densities of the cryo-ET map. Prior information about their assembly was used from the murine SD, as the resolution from the human SD alone was insufficient for a reliable fit. The arrangement of the heterodimers was selected so that no unoccupied densities remained. The spacings of the heterodimers between each other as well as to the membrane were according to the measurement of the cryo-ET map.

Sex as a biological variable: This study, although involving a sample obtained from a male patient, did not consider sex as a biological variable.
Statistics: 62 SD segments were combined to produce the final sub-tomogram average.

Study approval:
Ethics approval and consent to participate

Tissue samples used in this study were provided by the University Hospital Frankfurt. Written informed consent was obtained from the patient, and the study was approved by the institutional Review Boards of the UCT and the Ethical Committee at the University Hospital Frankfurt (project-number: SUG-6-2018).

Data availability: The cryo-ET data sets will be deposited to EMPIAR and to the Cryo-ET Data Portal.

References

7. Kelley K, et al. Waffle Method: A general and flexible approach for improving throughput in FIB-milling. *Nat Commun.* 2022;13(1):1857.
8. Burt A, et al. An image processing pipeline for electron cryo-tomography in RELION-5. *FEBS Open Bio.* 2024;14(11):1788-804.
9. Mastronarde DN, Held SR. Automated tilt series alignment and tomographic reconstruction in IMOD. *J Struct Biol.* 2017;197(2):102-13.
10. Kunz M, Frangakis AS. Three-dimensional CTF correction improves the resolution of electron tomograms. *J Struct Biol.* 2017;197(2):114-22.
11. Frangakis AS. Mean curvature motion facilitates the segmentation and surface visualization of electron tomograms. *J Struct Biol.* 2022;214(1):107833.
12. Ermel UH, et al. ArtiaX: An electron tomography toolbox for the interactive handling of sub-tomograms in UCSF ChimeraX. *Protein Sci.* 2022;31(12):e4472.
13. Meng EC, et al. UCSF ChimeraX: Tools for structure building and analysis. *Protein Sci.* 2023;32(11):e4792.
14. Emsley P, et al. Features and development of Coot. *Acta Crystallogr D Biol Crystallogr*. 2010;66(4):486-501.
15. Abramson J, et al. Accurate structure prediction of biomolecular interactions with AlphaFold 3. *Nature.* 2024;630(8016):493-500.

Supplementary figure 1

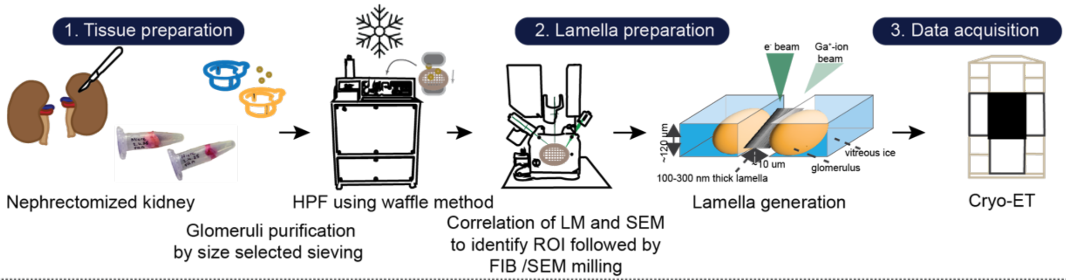

Supplementary figure 2

2D micrograph of the SD displays the strands spanning the foot processess

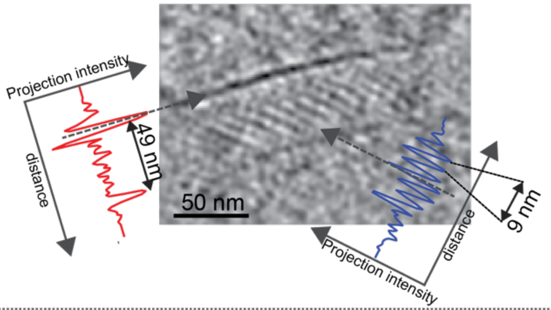

Supplementary figure 3

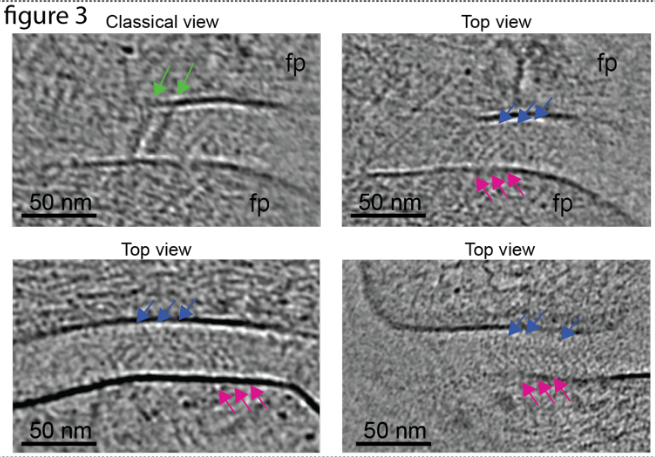

Supplementary figure 4

a Isosurface of the sub-tomogram averaging superimposed to the SD model

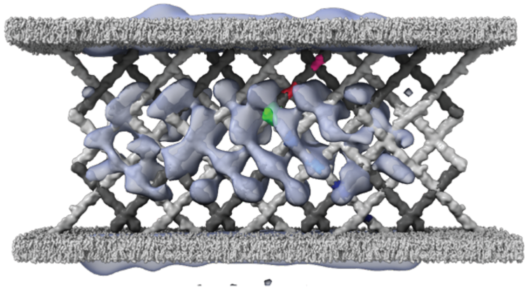

b Albumin in red in front of the SD model

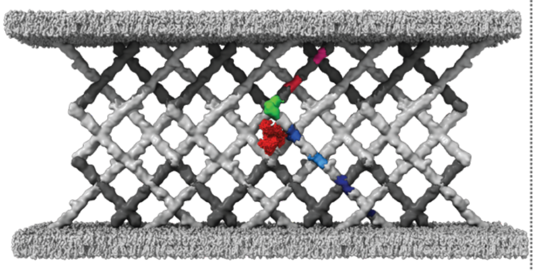

c

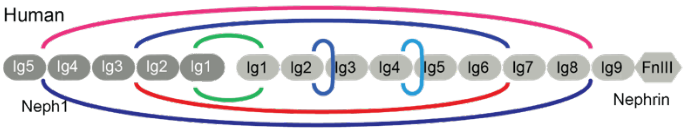

Supplementary figures:

Supplementary Figure S1: Glomeruli preparation workflow. Glomeruli were isolated from a nephrectomized human tissue by size-selected sieving and subjected to high-pressure freezing (HPF) to liquid nitrogen temperatures. A random area of high-pressure frozen sample was thinned to lamella of a thickness of ~250 nm with a focused-ion beam (FIB) scanning electron microscope (SEM). Finally, the lamellae were imaged using cryo-electron tomography (cryo-ET).

Supplementary Figure S2: Example of a 2D electron micrograph with a particularly strong contrast. The strands spanning the two foot processes can be seen at a spacing of ~9 nm. Because it is a 2D projection, only one layer of molecules is visible. The plots in the two directions display the spacing of the plasma membranes (in red) and the spacing of the strands (in blue).

Supplementary Figure S3: Four example of SDs seen in the cryo-electron tomograms. Computational sections through the SDs are shown (similar view to Figure 1c). The fishnet pattern with identical dimensions is visible in the noisy raw data. This signal was used for averaging and consequently improving the signal-to-noise ratio.

Supplementary Figure S4: Models of the SD. (a) The SD model superimposed on the isosurface of the sub-tomogram average (in transparent blue). One heterodimer is shown in different colors highlighting the crossing points. (b) Superposition of albumin shown in red (PDB:1AO6) in front of the schematic of the SD, showing their relative dimensions. The size of the structural holes compared to albumin can be appreciated. (c) Tentative interactions between Ig domains for the different organisms (*Drosophila*, Mouse, Human). The different Ig domains are schematically drawn as small rounded boxes. Connections in same colors as in the cartoon (Supplementary Figure 4) show the tentative interaction in the 3D model.
